# Managing Allergic Rhinitis in the Pharmacy: An ARIA Guide for Implementation in Practice

**DOI:** 10.3390/pharmacy8020085

**Published:** 2020-05-16

**Authors:** Olga Lourenço, Sinthia Bosnic-Anticevich, Elísio Costa, João A. Fonseca, Enrica Menditto, Biljana Cvetkovski, Vicky Kritikos, Rachel Tan, Anna Bedbrook, Sophie Scheire, Claus Bachert, Sławomir Białek, Vitalis Briedis, Koen Boussery, G. Walter Canonica, Tari Haahtela, Piotr Kuna, Ettore Novellino, Bolesław Samoliński, Holger J. Schünemann, Dana Wallace, Jean Bousquet

**Affiliations:** 1 Faculty of Health Sciences and CICS—UBI, Health Sciences Research Centre, University of Beira Interior, 6200-506 Covilhã, Portugal; 2 Woolcock Institute of Medical Research, Faculty of Medicine and Health, University of Sydney, Sydney 2037, Australia; sinthia.bosnic-anticevich@sydney.edu.au (S.B.-A.); biljana.cvetkovski@sydney.edu.au (B.C.); vicky.kritikos@sydney.edu.au (V.K.); rachel.sze.tan@sydney.edu.au (R.T.); 3 UCIBIO, REQUIMTE, Faculty of Pharmacy and Competence Center on Active and Healthy Ageing (AgeUPNetWork), University of Porto, 4150 Porto, Portugal; emcosta@ff.up.pt; 4 CINTESIS—Center for Health Technology and Services Research, Faculty of Medicine, University of Porto, 4200-450 Porto, Portugal; fonseca.ja@gmail.com; 5 CIRFF, Center of Pharmacoeconomics, University of Naples Federico II, 80138 Naples, Italy; enrica.menditto@unina.it; 6 MACVIA-France, 34295 Montpellier, France; anna.bedbrook@inserm.fr; 7 Pharmaceutical Care Unit, Faculty of Pharmaceutical Sciences, Ghent University, 9000 Ghent, Belgium; Sophie.Scheire@ugent.be (S.S.); koen.boussery@ugent.be (K.B.); 8 Upper Airways Research Laboratory, ENT Dept, Ghent University Hospital, 9000 Ghent, Belgium; claus.bachert@ugent.be; 9 International Airway Research Center, First Affiliated Hospital Guangzhou, Sun Yat-sen University, Guangzhou 510275, China; 10 Department of Medicine Solna, Immunology and Allergy Research Unit, Karolinska Institute, 171 77 Stockholm, Sweden; 11 Department of Biochemistry and Clinical Chemistry, Faculty of Pharmacy with the Division of Laboratory Medicine, Warsaw Medical University, 02-091 Warsaw, Poland; slawomir.bialek@wum.edu.pl; 12 Department of Clinical Pharmacy, Lithuanian University of Health Sciences, 44307 Kaunas, Lithuania; vitalis.briedis@lsmuni.lt; 13 Personalized Medicine Clinic Asthma & Allergy, Humanitas University, Humanitas Research Hospital, 20089 Rozzano MI, Italy; giorgio_walter.canonica@hunimed.eu; 14 Skin and Allergy Hospital, Helsinki University Hospital, University of Helsinki, 00100 Helsinki, Finland; tari.haahtela@haahtela.fi; 15 Division of Internal Medicine, Asthma and Allergy, Barlicki University Hospital, Medical University of Łódź, 90-419 Łódź, Poland; piotr.kuna@umed.lodz.pl; 16 Department of Pharmacy, University of Naples Federico II, 80138 Naples, Italy; ettore.novellino@unina.it; 17 Department of Prevention of Environmental Hazards and Allergology, Warsaw Medical University, 02-091 Warsaw, Poland; boleslaw.samolinski@wum.edu.pl; 18 Department of Health Research Methods, Evidence, and Impact, Division of Immunology and Allergy, McMaster University, Hamilton, ON L8S 4L8, Canada; holger.schunemann@mcmaster.ca; 19 College of Allopathic Medicine, Nova Southeastern University, Fort Lauderdale, FL 33314, USA; drdanawallace@gmail.com; 20 Centre Hospitalier Universitaire de Montpellier, 34295 Montpellier, France; jean.bousquet@orange.fr; 21 Charité—Universitätsmedizin Berlin, corporate member of Freie Universität Berlin, Humboldt-Universität zu Berlin, 10117 Berlin, Germany; 22 Comprehensive Allergy Center, Department of Dermatology and Allergy, Berlin Institute of Health, 10178 Berlin, Germany

**Keywords:** allergic rhinitis, community pharmacy, pharmacist

## Abstract

The paradigm of how we manage allergic rhinitis is shifting with a growing understanding that it is a complex process, requiring a coordinated effort from healthcare providers and patients. Pharmacists are key members of these integrated care pathways resolving medication-related problems, optimizing regimens, improving adherence and recommending therapies while establishing liaisons between patients and physicians. Community pharmacists are the most accessible healthcare professionals to the public and allergic rhinitis is one of the most common diseases managed by pharmacists. Allergic Rhinitis and its Impact on Asthma (ARIA) guidelines developed over the past 20 years have improved the care of allergic rhinitis patients through an evidence-based, integrated care approach. In this paper, we propose an integrated approach to allergic rhinitis management in community pharmacy following the 2019 ARIA in the pharmacy guidelines.

## 1. Introduction

Allergic rhinitis (AR) is the most common form of noninfectious rhinitis and is one of the most common chronic diseases globally [1,2,3,4]. Cardinal symptoms of AR include rhinorrhea, nasal congestion, sneezing and nasal itching, and they are often spontaneously reversible or controlled by adequate treatment [5]. These symptoms have a significant impact on work and school productivity [6,7], decreasing general health-related quality of life [8,9]. Clinical evaluation and the implementation of a clinical management plan that identifies specific risk factors and determinants of low adherence to treatment can effectively lead to symptom control and an improvement in the quality of life [5]. However, many patients underestimate their condition [10] or experience fatigue due to multiple suboptimal encounters with healthcare providers [11] and delay appropriate treatment. Moreover, self-medication for the treatment of AR symptoms is common [12], with most patients self-managing their AR with few interactions with their physician [13,14]. Furthermore, AR patients are often dissatisfied with their treatment and search for nonhealthcare professional advice for ways to manage their condition [11]. Finally, adherence to prescribed treatment in AR is low [15,16].

These considerations highlight the importance of effective, evidence-based advice and support being provided to AR patients at the community pharmacy level. The need for greater healthcare professional involvement is particularly important for people with other comorbidities, as AR is frequently associated with asthma and/or conjunctivitis [17]. Insufficient AR control can result in disease progression and affect asthma control [5]. Allergic Rhinitis and its Impact on Asthma (ARIA) guidelines, developed over the past 20 years, have improved the care of AR patients through an evidence-based, integrated care approach [5,18,19,20]. Specific guidelines for community pharmacies were first issued in 2004 [21] and have recently been updated to encompass integrated care pathways and digital solutions [22].

Initiated in 1999, in collaboration with the World Health Organization (WHO) [19], ARIA has developed into MASK (Mobile Airways Sentinel NetworK) [23,24,25], the IT solution of ARIA. In 2014, on behalf of the European Innovation Partnership on Active and Healthy Ageing (EIP on AHA), AIRWAYS ICPs (Integrated Care Pathways for airway diseases) was initiated [26,27]. The objective was to launch a collaboration to develop multisectoral care pathways (ICPs) for chronic respiratory diseases in European countries. MASK was proposed to be the mHealth strategy of AIRWAYS ICPs. MASK was developed as a proof-of-concept for multimorbid chronic disease care across the life cycle [28,29] and is scaled up using the EIP on AHA strategy [30,31]. It is a Good Practice of DG Santé [25,32] (digitally enabled, integrated, person-centred care for rhinitis and asthma) for change management [33]. MASK involves all stakeholders including professionals and patients, as well as policymakers and healthcare authorities.

It is now critical to put these guidelines into practice in the community pharmacy setting. AR is a complex chronic condition, and implementing clinical practice guidelines for AR management in the community pharmacy is particularly important, as most patients do not consult a medical practitioner when selecting medication for their AR [5,20,34]. The pharmacist’s role must, therefore, encompass a broad range of management issues including confirmation of the presence of AR, treatment selection, patient self-management, long-term monitoring and patient support.

## 2. Is this Allergic Rhinitis?

With the high level of self-diagnosis, the ability of pharmacists to identify AR is critical. In an Australian study of patients with moderate asthma, 46% of those who also had AR did not have a physician diagnosis [14]. Moreover, in a study in Australian community pharmacies, 37% of patients purchasing AR medication did not have a physician diagnosis [35]. To confirm the presence of AR, the pharmacist needs to verify the patient’s symptoms and exclude alternative conditions such as the common cold or other more severe illnesses [36] which may require immediate referral to a physician [37,38]. The possibility of drug-induced rhinitis (e.g., nonsteroidal anti-inflammatory drugs, alpha-antagonists and alpha-2 agonists) should be assessed, as medication adverse effects will not respond to therapy. Table 1 provides a set of simple questions that may help the pharmacist in differentiating allergic-rhinitis-like symptoms from other causes.

AR patients may have multiple nasal symptoms (rhinorrhea, sneezing, nasal pruritus and/or congestion), a large percentage have ocular symptoms (ocular tearing, redness and pruritus) and many patients also have asthma [39]. Although nasal congestion is an AR symptom, it is unlikely to be of allergic origin when it occurs as a single symptom. The issue may be, however, that patients do not necessarily realise that they are experiencing more than one symptom [35]. Loss of smell, facial pain or postnasal drip are more often associated with nonallergic rhinitis [40] or rhinosinusitis. Patients presenting with unilateral symptoms, recurrent nasal bleeding, clear rhinorrhea which is largely unilateral and worse on bending forward, or purulent rhinorrhea, especially if accompanied with fever, should be referred to a physician [38].

**Figure 1:**
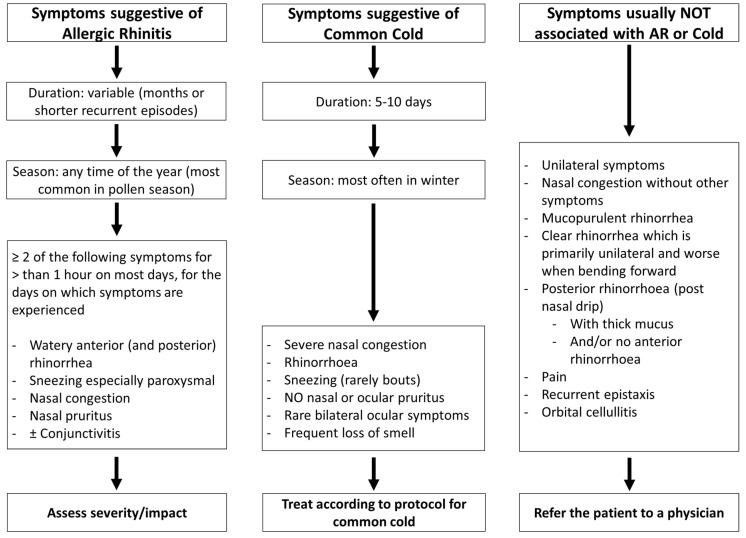
Recognising allergic rhinitis in the pharmacy (adapted from [21,22]). In Figure 1, some of the items not associated with allergic rhinitis symptoms refer to rhinosinusitis and other diseases that need to be checked during differential diagnosis.

Ocular symptoms are commonly associated with nasal symptoms in AR [41] and can be evaluated using simple questions. However, some forms of conjunctivitis require a referral to a physician, especially if they are unilateral, or if photophobia, bleeding or a burning sensation are present (Figure 1).

**Figure 2:**
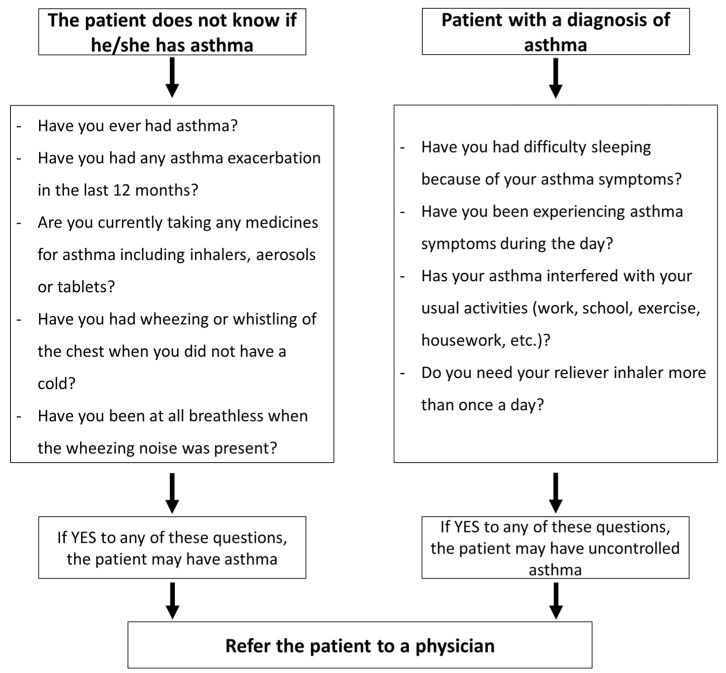
Screening of asthma in rhinitis patients in the pharmacy (Adapted from [22]).

The co-existence of poorly controlled rhinitis is associated with poor asthma control in adults, adolescents and children [42,43,44] and is a major risk factor for asthma exacerbations. If the patient presents asthma symptoms in addition to rhinitis symptoms, they should be referred to a physician (Figure 2). Therefore, it is critical to consider asthma in the evaluation of AR.

## 3. Assessing the Severity of Allergic Rhinitis: Tools for the Pharmacist

The ARIA guidelines propose a classification of AR based on symptom control, quality of life and daily impact as well as duration [5,18,20]. Allergic rhinitis may be intermittent or persistent, but this does not impact on the treatment to be recommended except for its duration. The ARIA guidelines base treatment recommendations on the impact of symptoms on day-to-day living [22,25,45].

Computer-based pharmacy systems have been used globally for decades, but they refer mainly to drug-drug interactions, adverse effects and drug allergy [46,47,48,49]. In 2012, a computational pharmacy decision support system (PDSS) for allergic rhinoconjunctivitis was tested in German community pharmacies. Its usefulness was proved in patient assessment and correct evaluation before self-medication counselling [50]. A newly developed computerized decision support system (e-CDSS) might be useful in identifying patients with a likelihood of having AR and in proposing the most effective treatment [51].

**Figure 3:**
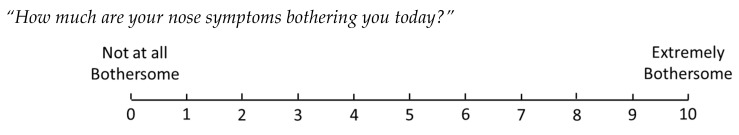
Determining the impact of allergic rhinitis symptoms.

Symptom severity in AR can be measured using total symptoms score, but this is not a simple test. The visual analogue scale (VAS) is a simple, validated quantitative measure, which can be used in a wide variety of languages and which is responsive to change [52]. The VAS uses patient-reported assessments of the intensity of the main symptoms. A VAS for nasal symptoms (range 0–10) is proposed in the treatment flowchart (Figure 3) to assess control before and after some days of treatment. A VAS for ocular symptoms may also be used and can complement the VAS for nasal symptoms [53].

*“How much are your nose symptoms bothering you today?”*

For patients with simultaneous asthma and rhinitis, a previous study conducted in community pharmacies showed that a simple self-assessment questionnaire, the Control of Allergic Rhinitis and Asthma Test (CARAT) [54], can help the pharmacist to identify patients with uncontrolled AR and asthma, which is the first step in improving symptom control [55].

## 4. Treatment of Allergic Rhinitis in the Pharmacy

Minimizing the impact of AR symptoms is the most important endpoint for the pharmacist, and is used to initiate treatment and maintain appropriate medication/strategies over time. AR symptoms can be used to evaluate the efficacy of the treatment and the patient’s quality of life. By evaluating the impact of AR on day-to-day living, a VAS (as per Figure 3) can be used. While a majority of AR patients self-select their medication, the role of the pharmacist is crucial as AR patients who obtain professional advice from a pharmacist have a higher chance of choosing guideline-recommended medication [37].

AR treatment encompasses three different aspects—(1) avoidance of allergen exposure; (2) pharmacotherapy; and (3) allergen-specific immunotherapy (AIT). Pharmacists have an opportunity to deliver patient education in terms of avoidance of allergen exposure, disease information, as well as medication recommendations and use, including nasal medication administration and adherence [45].

(1) With regard to allergen avoidance and environmental control, these strategies can diminish AR symptoms, reducing the need for medication [18]. However, this approach is only feasible when allergens have been identified, and its exposure can be reduced effectively; which is nearly impossible for polysensitized patients and patients with pollen allergy during the respective pollen season [56]. Nevertheless, the use of the pollen app should be suggested to patients if available for the geographic region.

(2) When it comes to medications, guidelines consider a range of medications that can be used in the treatment of AR, based on symptom severity, duration and on the impact of symptoms on day-to-day living [5,34,57,58,59,60]. Medications primarily include intranasal corticosteroids (INCS), intranasal and oral antihistamines, leukotriene antagonists, intranasal cromoglicate, intranasal and oral vasoconstrictors and nasal washing. It is important to note that medication lists vary widely between countries.

(3) AIT helps to reduce the severity of AR symptoms by modifying the immunological response and inducing tolerance to the causative agent [45]. Products for AIT are available in pharmacies of many countries and the pharmacist can be an important participant in the management of AIT, providing monitoring for adverse effects and support for adherence [45].

INCS are the most effective treatment for AR, especially in patients with co-existing asthma [19,34,61] and are considered nonprescribed medication in many countries. All of the available intranasal corticosteroids are efficacious in controlling symptoms, although mometasone furoate, fluticasone propionate and fluticasone furoate are generally preferred [62] because they have negligible bioavailability and less potential to cause side effects. Pricing, cultural barriers, specific country regulations and availability, and even patient preference [63,64] for oral vs. nasal treatment all mean that INCS may not necessarily be available or the most desirable treatment for all patients, such as those with a predisposition to high intraocular pressure (glaucoma). Oral H_1_-antihistamines are still largely used by many patients and may be sufficient to control mild AR. The intranasal combination of azelastine and fluticasone propionate is available OTC in New Zealand. Therefore, a broad understanding of the local context should be taken into consideration when recommending treatment.

Studies reporting interventions targeting nasal inhaler medication administration techniques for AR management are missing. However, the results of bronchial inhaler community-pharmacy interventions used for COPD and asthma have been encouraging [65,66,67,68]. As such, patients should be counselled and evaluated on the appropriate administration technique for intranasal medication, especially in avoiding the nasal septum. In avoiding the nasal septum, medication efficacy is increased and the risk of local adverse effects such as nasal crusting and bleeding is reduced which is more likely to lead to improved compliance to treatment [69].

Some INCS reduce eye symptoms as well as nasal symptoms, although the combination of INCS and ocular antihistamines is more effective on both nasal and ocular symptoms [70,71,72]. Moreover, there are topical drugs for eye symptoms [18].

However, none of the medications currently used to treat AR can cure this condition and many patients still suffer from uncontrolled disease either because they are not adherent to treatment or medications cannot fully control the disease [36]. As such, the pharmacist needs to help patients self-manage their condition by determining the most effective treatment to control their symptoms from the options available, with consideration of their needs and preferences. In AR, the main goal of treatment should be improving the patient’s wellbeing and daily functioning. In some selected AR patients, allergen-specific immunotherapy can be a valuable alternative.

**Figure 4:**
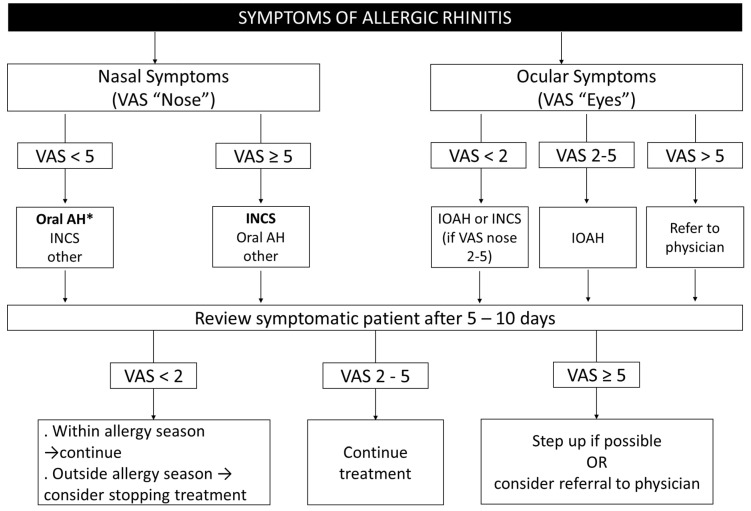
Treatment of allergic rhinitis in the pharmacy. AH, antihistamine; INAH, intranasal antihistamine; INCS, intranasal corticosteroid; IOAH, intraocular antihistamine. *INCS if coexisting asthma (adapted from [22]).

Figure 4 presents a flowchart based on VAS to help pharmacists determine which medication is indicated for individual patients experiencing AR symptoms. This algorithm should be adapted to the regulations, needs, price of medications and cultural barriers of each country or region.

VAS nose: *“How much are your nose symptoms bothering you today?”*

VAS eyes: *“How much are your eye symptoms bothering you today?”*

## 5. Counselling on Possible Side Effects of Medications

Antihistamines and decongestants are frequently used for the treatment of AR [73]. However, pharmacists fulfil a key role in the selection of appropriate self-treatment by patients in the light of possible side effects. Many nonprescribed medications contain first-generation H1 antihistamines (e.g., clemastine, cyproheptadine, hydroxyzine, ketotifen, promethazine) that should be avoided because of their anticholinergic and sedating effects and the related risk of, for example, road accidents [74,75]. Oral decongestants should also be used with caution due to possible side effects, including insomnia, elevated blood pressure, and tachycardia [61].

Intranasal decongestants can be used for short-term and possibly episodic treatment of nasal congestion, but prolonged use may lead to rhinitis medicamentosa (RM) [76]. RM is a form of drug-induced, nonallergic rhinitis caused by the excessive or improper use of topical decongestants. As a result, the patient ends up in a vicious cycle of self-treatment to alleviate nasal congestion [77,78].

A community pharmacy-based study has indicated that overuse of nasal decongestants is prevalent in patients self-medicating persistent rhinitis [79]. Therefore, recommending brief use of fewer than 10 days by the dispensing pharmacist is crucial in the prevention of RM [77,78,80]. Symptoms of RM include long-term redness and swelling of the nasal mucosa and increased runny nose. If this occurs, the patients should be instructed to stop using the medication and consult a physician or a pharmacist.

## 6. Patient’s Self-Management of AR and Patient Support

Self-management plays a particularly important role in the management of AR and is a major challenge for four key reasons—(1) patients often underestimate their symptoms; (2) trivialize their condition; (3) do not adhere to medication; and (4) fail to review treatment to assure that it is working [37,55]. Moreover, there is increased access to medications, especially for long-established prescription drugs with good safety profiles that have been rescheduled as OTC [81]. Furthermore, in some countries, OTC medications may be obtained without the intervention of a pharmacist [37]. Patients should also be educated on when to expect symptom relief. In the case of INCS, the benefit may take up to two days to be clinically evident and several days to be fully effective [82].

AR has a significant impact on individuals, creating an important economic burden with substantial direct (expenditure for medication, hospitalizations, access to medical care) and indirect (absenteeism, presenteeism, decreased school/work performance) costs [83]. Appropriate/optimal self-care and self-management of chronic conditions have been shown to improve adherence, improve disease outcomes and reduce the burden on health services [84,85,86,87]. An important aspect of this self-management is the input of healthcare professionals. Healthcare professional-directed goals and strategies in self-management result in better clinical outcomes than goals and strategies derived from personal choice only [11,37,38,88]. Patients need to have their AR and treatment reviewed over time with a healthcare professional.

mHealth tools have conquered the general public and an enormous number of monitoring applications, that are freely available, user friendly and intuitive are currently being used. Self-management, outside the confines of formal healthcare, but supported on mHealth tools, may ensure that the patient is properly informed and educated about his/her disease and can apply a course of action to ensure optimal outcomes [89].

Mobile technology may help to better understand the low adherence to treatment in allergic diseases and asthma [90,91]. Many mobile phone apps are available to support people in taking their medications and to improve medication adherence [92,93]. However, the majority of them do not have many of the desirable features and are of low quality [93].

Adherence is a dynamic phenomenon and optimal adherence is crucial for treatment success. Community pharmacy-led interventions can improve patient adherence to medications and contribute to better COPD and asthma control [67,94]. To the best of our knowledge, no community pharmacy interventions designed specifically to improve AR medication adherence have been published. However, the literature already available in other settings supports the use of educational interventions in the community pharmacy [94].

MASK-air is a very usable app, specific for AR monitoring which has been validated with published results, being implemented in 23 countries and 17 languages [23,24,95,96]. This tool is appropriate for most patients and enables patient follow-up. In addition to being a tool to assist patients, MASK-air can also be used to discuss AR with healthcare providers through the interoperable application MASK-air Companion [97]. Using MASK-air, a patient is able to print out the daily AR symptoms and share them with the pharmacist (or other healthcare providers). The MASK-air app also encompasses questions related to symptoms, quality of life and work, with a simple interface using VAS [98] and the CARAT questionnaire [23].

## 7. Conclusions

The integration of all of the stakeholders proposed by ARIA in a common integrated pathway can improve disease outcomes [25,28]. Given the breadth of individuals and resources with whom AR patients are engaged [11], it is critical that an integrated care pathway approach is applied to the management of AR. Shared responsibility among patients, pharmacists, primary care/general practitioners and specialists can ensure appropriate, safe and cost-effective medication use, and lower healthcare utilization rates. The development of this integrated pathway in which the pharmacist is a member of the interdisciplinary team can affect the quality of both the individual healthcare services and the patient’s healthcare plan. Moreover, what truly distinguishes the ARIA proposal is that it considers the patient’s needs and desired outcomes for their disease and quality of life.

## Figures and Tables

**Table 1:** Questions helping to identify allergic rhinitis and/or allergic conjunctivitis.

Questions Helping to Identify Allergic Rhinitis
What is your main symptom? (Check for rhinorrhea, sneezing, itchy nose, nasal congestion, watery or itchy eyes.)
How long have you had these symptoms?
Do you have the symptoms all the time or do they come and go?
Are you aware of anything that seems to bring the symptoms on, such as being outdoors, pollen seasons, contact with animals, something you handle at work or at home?
Has a doctor ever diagnosed you with hay fever, allergic rhinitis, or asthma?
Is your nasal discharge clear and watery? (purulent discharge suggests infection)
Are you experiencing any wheezing or shortness of breath? (“Yes” may indicate asthma.)
Do you have an earache or any pain in your face? (“Yes” may indicate otitis media or sinusitis.)
**Questions helping to identify allergic conjunctivitis**
What is your main symptom? (Check for bilateral eye symptoms, eye itching, watery eyes, red eyes)
Do you have allergic rhinitis?
Do your eyes burn? (“Yes” may indicate disease other than allergic rhinitis)
Do you have dry eyes? (“Yes” may indicate disease other than allergic rhinitis)
Do you have photophobia? (“Yes” may indicate disease other than allergic rhinitis and the patient should be referred to a doctor)
